# Prolactin, cortisol and heat shock proteins in early sepsis: preliminary data

**DOI:** 10.1186/cc13424

**Published:** 2014-03-17

**Authors:** K Vardas, K Apostolou, K Psarra, E Botoula, S Tsagarakis, E Magira, C Routsi, E Briassouli, D Goukos, S Nanas, G Briassoulis

**Affiliations:** 1 Medical School, NKUA, Athens, Greece; 2 Evangelismos Hospital, Athens, Greece; 3 University Hospital, University of Crete, Heraklion, Greece

## Introduction

Besides cortisol, prolactin might also be important in maintaining normal immune response in stress states. Its role in the hypothalamus-pituitary-adrenal axis hormonal and immune stress response in sepsis has hardly been investigated in a critically ill setting. The aims of the study were to evaluate the early (first 48 hours) ACTH, cortisol and prolactin plasma levels in ICU patients with severe sepsis/ septic shock (SS) or systemic inflammatory response syndrome (SIRS) compared with healthy control subjects (C) and to correlate their expression with heat shock proteins (HSPs), interleukins (ILs) and outcome.

## Methods

Thirty consecutively admitted patients with SS, 22 with SIRS, and 15 C were enrolled in the study. Patients' demographics, laboratory examinations, and APACHE II, SOFA and SAPS III scores were recorded on ICU admission. Blood sampling was performed between 9:00 and 10:00 a.m. Prolactin and cortisol were measured using the ADVIA centaur system, ACTH using the Immulite 2000. Intracellular monocyte HSP (iHSP) was determined after staining with surface antigens CD33- PE/Cy5 and CD45 PE/Cy7 followed by either HSP72-FITC or HSP90a-PE intracellular staining (four-color flow cytometry). Mean fluorescence intensity values for each HSP were noted and analyzed. ELISA was used to determine extracellular (e) HSP, IL-6 and IL-10 levels.

## Results

Prolactin (*P *< 0.034), cortisol (*P *< 0.0001), and ACTH (*P *< 0.02) levels differed among groups. Prolactin, cortisol, IL-6, and eHSP90 were higher in SS (*P *< 0.03) and SIRS (*P *< 0.04) compared with C. Cortisol and eHSP90 were higher in SS compared with SIRS (*P *< 0.005; *P *< 0.03); iHSP72 and iHSP90 were higher in SIRS compared with SS (*P *< 0.05; *P *< 0.05) or C (iHSP72, *P *< 0.02). Cortisol related with eHSP90 (*r *= 0.45, *P *< 0.02), lactate (*r *= 0.33, *P *< 0.02) and HCO^3 ^(*r *= -0.45), and prolactin with SAPS III (*r *= 0.34, *P *< 0.02), but not with mortality or LOS.

## Conclusion

Prolactin seems to be a stress hormone, probably related to the severity of illness, increased along with IL-6 and eHSP90 levels in SS. Also in these patients, cortisol correlates to lactate and eHSP90, but in contrast to SIRS the iHSP72 and iHSP90 immune response is depressed.

## Acknowledgements

This research has been co-financed by the European Union (European Social Fund) and Greek national funds through the Operational Program 'Education and Lifelong Learning' of the National Strategic Reference Framework Research Funding Program: THALES.

